# PERK controls bone homeostasis through the regulation of osteoclast differentiation and function

**DOI:** 10.1038/s41419-020-03046-z

**Published:** 2020-10-13

**Authors:** Jiachao Guo, Ranyue Ren, Kai Sun, Xudong Yao, Jiamin Lin, Genchun Wang, Zhou Guo, Tao Xu, Fengjing Guo

**Affiliations:** 1 Department of Orthopedics, Tongji Hospital, Tongji Medical College, Huazhong University of Science and Technology, 1095 Jiefang Avenue, 430030 Wuhan, China; 2 Department of Rehabilitation, Tongji Hospital, Tongji Medical College, Huazhong University of Science and Technology, 1095 Jiefang Avenue, 430030 Wuhan, China

**Keywords:** Bone, Metabolic disorders

## Abstract

Osteoclasts are multinucleated giant cells with the ability to degrade bone tissue, and are closely related to abnormal bone metabolic diseases. Endoplasmic reticulum (ER) is an organelle responsible for protein modification, quality control, and transportation. The accumulation of unfolded or misfolded proteins in ER cavity induces ER stress. Double-stranded RNA-dependent protein kinase-like ER kinase (PERK) is an ER stress-sensing protein, which is ubiquitous in eukaryotic cells. Systemic PERK knockout mice show severe bone loss, suggesting that PERK is of great significance for maintaining the normal growth and development of bone tissue, but the role of PERK in osteoclastogenesis is still unclear. In this study, we found that PERK was significantly activated during RANKL-induced osteoclast differentiation; knockdown of PERK by siRNA and inhibition of PERK by GSK2606414, respectively, had significant negative regulatory effects on the formation and bone resorption of osteoclasts. PERK inhibitor GSK2606414 down-regulated the mRNA levels and protein expression of osteoclast differentiation marker genes, and inhibited RANKL-induced activation of Mitogen-activated protein kinase (MAPK) and nuclear factor κB (NF-κB) pathways. Treatment with PERK inhibitor GSK2606414 in ovariectomized mouse model significantly suppressed bone loss and osteoclast formation. Thapsigargin activated ER stress to enhance autophagy, while GSK2606414 had a significant inhibitory effect on autophagy flux and autophagosome formation. Antioxidant *N*-acetylcysteine (NAC) could inhibit the expression of PERK phosphorylation, osteoclast-related proteins and autophagy-related proteins, but the use of PERK activator CCT020312 can reverse inhibition effect of NAC. Our findings demonstrate a key role for PERK in osteoclast differentiation and suggest its therapeutic potential.

## Introduction

Bone is a continuously renewing organ and maintains dynamic balance which was regulated through osteoclasts and osteoblasts to meet the needs of injury repair and growing development^1^. Osteoclasts are multinucleated giant cells originated from monocyte-derived macrophages, and own vital ability to resorb bone^2^. Excessive activation or dysfunction of osteoclasts is associated with many diseases, such as postmenopausal osteoporosis, periodontitis, rheumatoid arthritis, and Paget’s disease^3^. Osteoclast differentiation and function are mainly regulated by two cytokines: receptor activator of nuclear factor κB ligand (RANKL) and macrophage-colony stimulating factor (M-CSF)^4^. M-CSF could combines with macrophage-colony stimulating factor receptor (M-CSFR) to promote proliferation and survival of osteoclast precursors, and regulate persistent expression of receptor activator of nuclear factor κB (RANK)^5^. RANKL binds to RANK on the surface of osteoclast precursor cells, recruiting the adaptor molecules, especially TRAF6. Then RANKL-RANK-TRAF6 complex is formed and activates downstream signaling pathways including NF-κB, MAPKs (ERK, JNK, and p38), AP-1 and NFATc1, which together promote differentiation and function of osteoclast^6–8^.

The endoplasmic reticulum (ER) is an organelle responsible for protein synthesis, modification, and quality control. Various pathological stimuli such as nutritional deprivation, oxidative stress, ER calcium depletion, altered glycosylation, and energy perturbations, may disrupt intracellular homeostasis and cause accumulation of unfolded or misfolded proteins in the ER cavity, which leads to ER stress^9,10^. ER stress is associated with the inflammatory process. Rheumatoid arthritis (RA), which is characterized by excessive osteoclast activity, is thought to have a close relationship between its pathogenesis and ER stress^11,12^. Hypoxia/ischemia injury is observed in the synovium of RA patients, which may increase the amount of unfolded proteins in ER cavity of synovial cells^13^. Unfolded protein response (UPR) is an evolutionarily conserved pathway of ER stress, and it contains three key transmembrane proteins, activating transcription factor 6 (ATF6), inositol-requiring enzyme 1 (IRE1) and double-stranded RNA-dependent protein kinase-like ER kinase (PERK)^14^. They can mediate the corresponding pathways to inhibit protein synthesis and promote the degradation of unfolded proteins, so as to alleviate ER stress and restore ER function^15^. As an ER stress sensor protein that is ubiquitous in eukaryotic cells, PERK is essential for regulating the collagen and growth factors secreted by osteoblasts to maintain the normal growth and development of bone tissue^16^. Recent studies have found that *Perk* mutations can cause Wolcott-Rallison syndrome (WRS), which is mainly characterized by type I diabetes, mental retardation and multiple epiphyseal dysplasia occurring in the newborn or infant^17^. PERK is closely related to bone remodeling, but how to regulate osteoclast differentiation and function is still unclear.

Autophagy is an adaptive catabolic process of disassembling cellular components that exists in eukaryotic cells^18^. Recent studies have found that autophagy-related proteins are important regulators of osteoclast differentiation and function^19,20^. Both autophagy inhibitors 3-Methyladenine and chloroquine can effectively down-regulate the expression of osteoclast-related genes and inhibit osteoclastogenesis and its function^21,22^. Silencing the autophagy-associated gene Beclin1 in osteoclast precursors inhibits osteoclast differentiation and maturation. The possible mechanism is that it down-regulates RANKL-mediated NFATc1 expression and reduces mitochondrial ROS production^23^. Autophagy plays a key role in reducing the abnormal accumulation of proteins in the cell through the way of lysosome degradation of intracellular materials. Klionsky et al. found that ER stress can activate the autophagy pathway in yeast^24^. Studies have shown that PERK is involved in the transcriptional upregulation of many autophagy-related genes, including LC3, ATG12, ATG5, ULK, and Beclin1^25–27^.

In this study, we explored the effects of PERK on osteoclast differentiation and the role of autophagy in this process, and investigated the impact of PERK inhibitors on bone loss through in vitro model.

## Results

### ER stress activator thapsigargin promoted osteoclastogenesis, while GSK2606414 reversed its effect

**Fig. 1:**
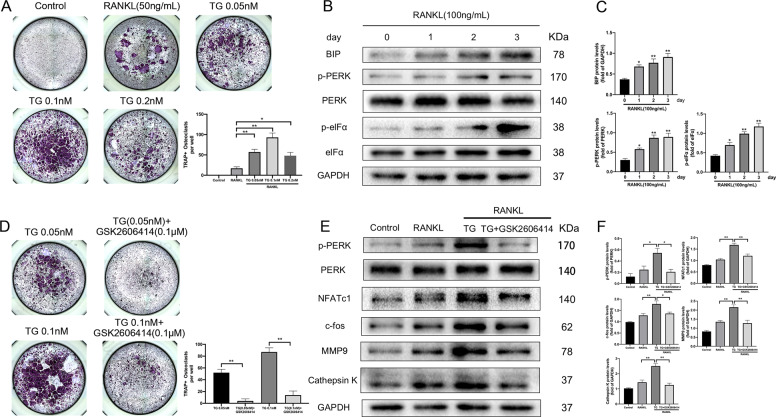
PERK pathway is activated during RANKL-induced osteoclast differentiation, and GSK2606414 inhibits the effect of thapsigargin on promoting osteoclastogenesis. **a** BMMs were cultured with RANKL (50 ng/mL) and various doses of thapsigargin (0, 0.05, 0.1, 0.2 nM) for 4 days, TRAP staining was conducted and TRAP-positive osteoclasts (≥3 nuclei) were counted. **b**, **c** RANKL (100 ng/mL) was used to stimulated BMMs differentiating into osteoclasts. Total proteins were extracted at different time points from 0 to 3 days of induction and western blot was performed to detect the proteins of PERK pathway. Densitometric analysis of an immunoblot from three independent experiments. *p < 0.05, **p < 0.01 versus 0 day group. **d**–**f** Thapsigargin and GSK2606414 were added to osteoclast-induced differentiation medium containing RANKL (50 ng/mL), respectively. TRAP staining was performed 4 days later and osteoclasts were counted. Total protein was extracted on day 4 and detect the protein expression of p-PERK, PERK and osteoclast differentiation marker genes. Densitometric analysis of an immunoblot from three independent experiments. *p < 0.05, **p < 0.01. TG represents thapsigargin in the figure.

To elucidate the role of ER stress in regulating osteoclast differentiation, we treated BMMs with thapsigargin, a classic activator of ER stress, and tested its effect on osteoclastogenesis. CCK-8 showed that 0.05–0.2 nM thapsigargin did no impact the viability of BMMs (Fig. S1A). Figure 1a exhibited that 0.05 nM and 0.1 nM thapsigargin significantly promoted osteoclast formation, while 0.2 nM thapsigargin had no significant effect on osteoclastogenesis. The results indicated that proper activation of ER stress could promote osteoclast differentiation. We further examined the expression of PERK pathway in the case of RANKL-induced osteoclast differentiation. As shown in Fig. 1b, c, the expression of BIP gradually increased with time, and accompanied by increased levels of p-PERK and p-eIFα. These results suggested that PERK pathway was apparently activated during osteoclast differentiation, which means that the ER stress-related PERK pathway may have played an important role in osteoclastogenesis.

Thapsigargin induces ER stress activation mainly by activating PERK and IRE1 pathways^28^, the small molecule compound GSK2606414 can bind to the PERK kinase domain and inhibit PERK activation by down-regulating PERK autophosphorylation^29^. We used GSK2606414 to verify whether PERK pathway plays a vital regulatory role in ER stress-induced osteoclastogenesis caused by thapsigargin. CCK-8 assay revealed that 0.01–0.1 μM GSK2606414 had no obvious inhibitory effect on BMMs proliferation (Fig. S1B). Figure 1d proved that GSK2606414 significantly suppressed the pro-osteoclastogenesis effect of thapsigargin. NFATc1 and c-fos are key transcription factors that regulate osteoclast differentiation, which regulate the expression levels of Cathepsin K and MMP9^7,30^. We found that thapsigargin could obviously increase the protein levels of p-PERK, NFATc1, c-fos, MMP9, and Cathepsin K during osteoclast differentiation. After treating with GSK2606414, the expression of these proteins were significantly decreased (Fig. 1e, f).

### Inhibition of PERK suppressed osteoclast differentiation and function

**Fig. 2:**
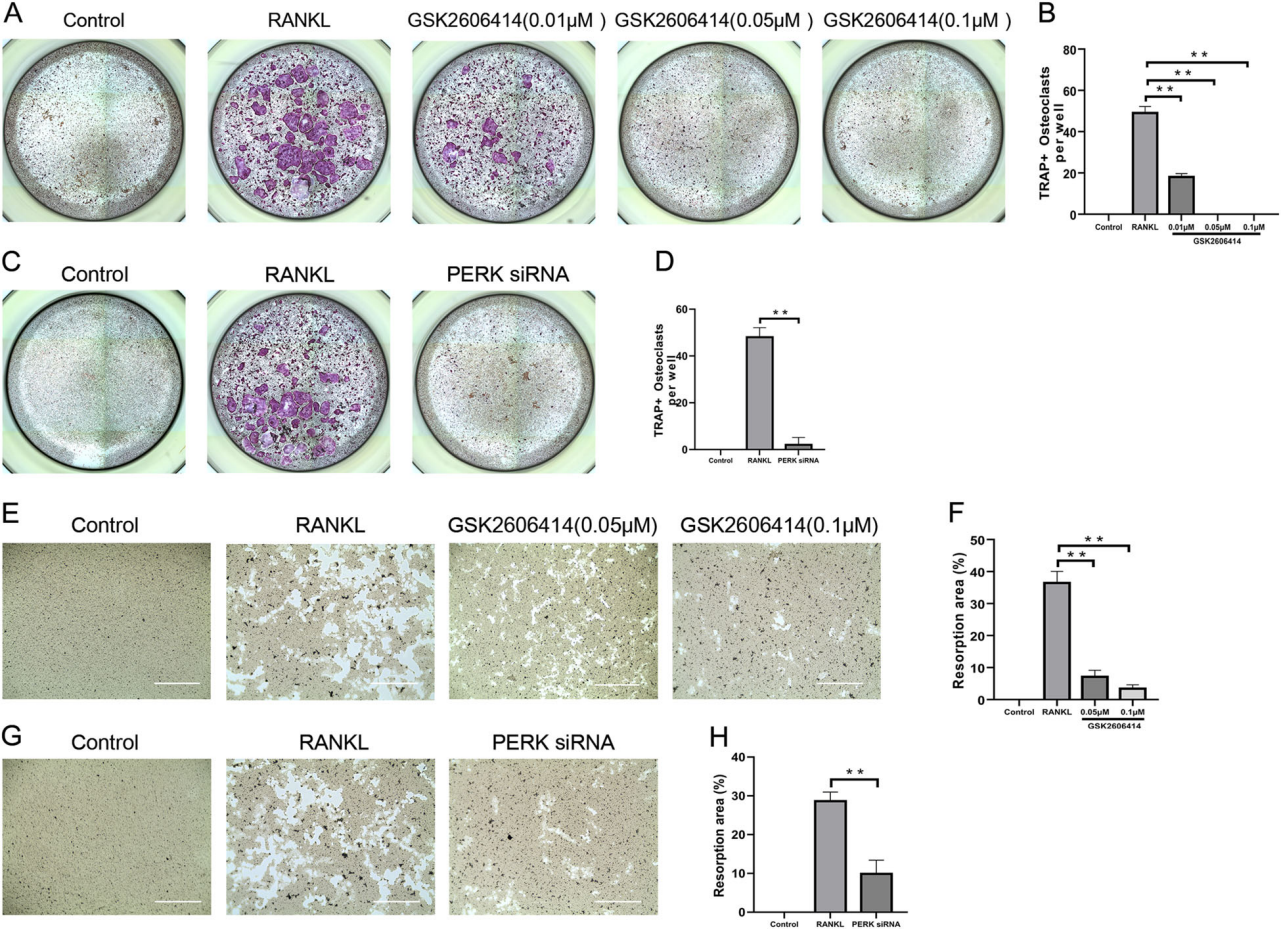
Inhibition of PERK suppresses osteoclast differentiation and bone resorption. **a**–**d** BMMs were treated with PERK siRNA or small molecule inhibitor GSK2606414 (0.01, 0.05, 0.1 μM) in the presence of RANKL (100 ng/mL) and M-CSF (30 ng/mL) for 4 days. Then, TRAP assay was performed. TRAP-positive multinucleated osteoclasts (≥3 nuclei) were counted. **e**–**h** BMMs were seeded in Osteo Aaasy Surface plate at a density of 2 × 10^4^ cells/well, and cultured with 100 ng/mL RANKL and 30 ng/mL M-CSF. After mature osteoclasts were formed in each group, PERK siRNA and GSK2606414 were applied for 3 days. Bone resorption area was quantified by using Image J, Scale bar = 400 μm. Data are presented as means ± SD of 3 independent experiments; **p < 0.01.

In order to explore the regulatory role of PERK in osteoclast differentiation, we used GSK2606414 and siRNA to achieve inhibition and silencing of PERK. After 4 days of RANKL-induced culture, TRAP staining was performed to observe the purple multinucleated giant cells (osteoclasts). By using GSK2606414 and siRNA, the number of mature osteoclasts formed was significantly lessened (Fig. 2a–d). This result indicated that inhibiting or silencing PERK had a strong inhibitory effect on osteoclastogenesis.

**Fig. 3:**
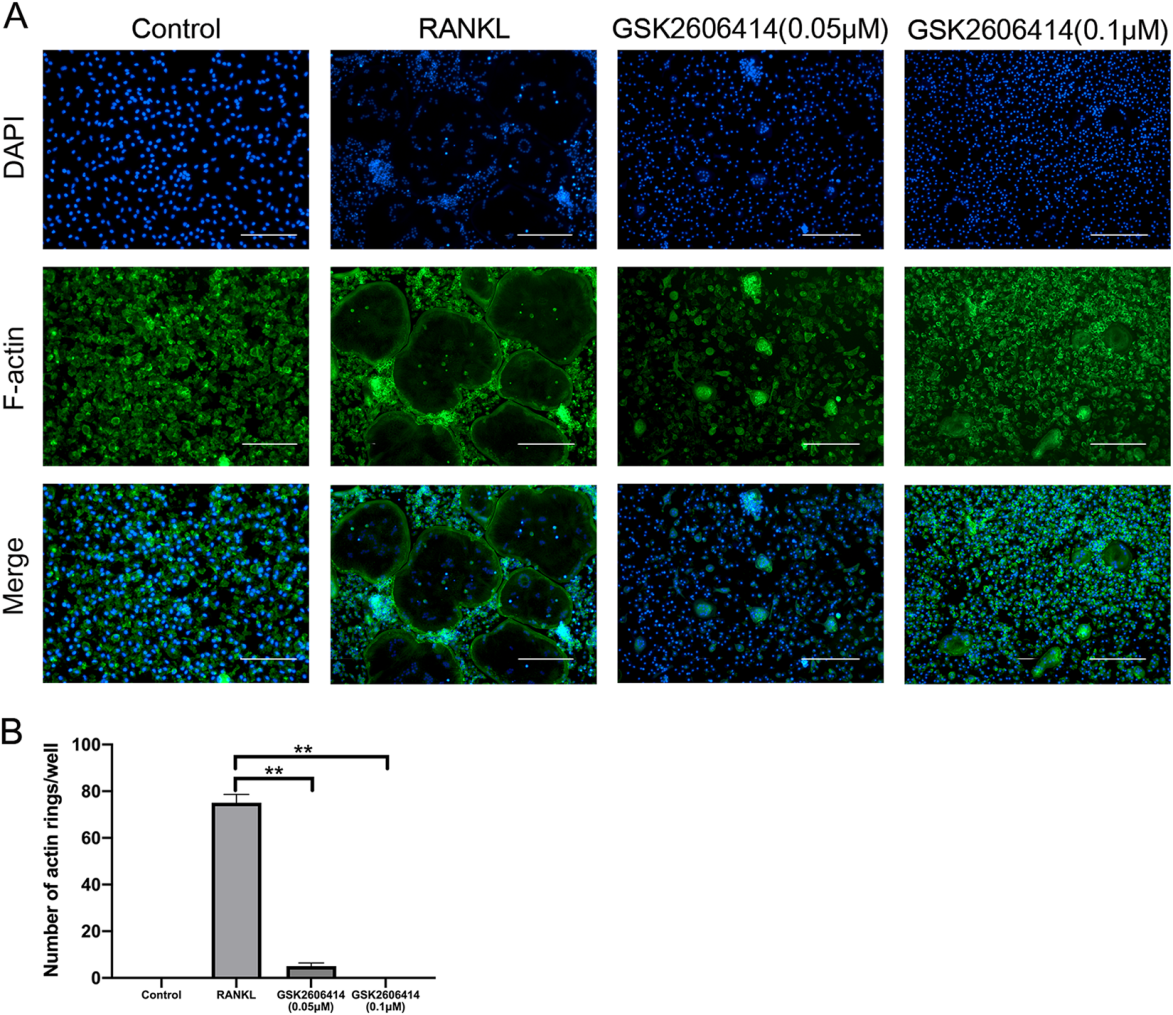
GSK2606414 suppresses osteoclast F-actin ring formation. BMMs were cultured with RANKL (100 ng/mL) and various concentrations of GSK2606414 (0.05, 0.1 nM) for 4 days, and actin ring fluorescence staining was performed. **a**, **b** F-actin ring fluorescence images were acquired and quantified. Scale bar = 400 μm, Data are presented as means ± SD of 3 independent experiments; **p < 0.01.

Bone slice resorption assays exhibited that GSK2606414 and PERK-siRNA significantly inhibited osteoclast function (Fig. 2e–h). The bone resorption function of osteoclasts starts from the “closed area” formed between cell membrane and the attachment surface, this part is rich in F-actin, and F-actin ring is the marker cytoskeleton structure after osteoclasts turning mature^31^. Consistent with the results of bone resorption assay, GSK2606414 and siRNA could significantly reduce the number of F-actin rings (Fig. 3a, b). In summary, PERK was a key regulatory factor in osteoclast differentiation and function.

### GSK2606414 inhibited expression of key transcription factors and related marker genes in osteoclast differentiation

**Fig. 4:**
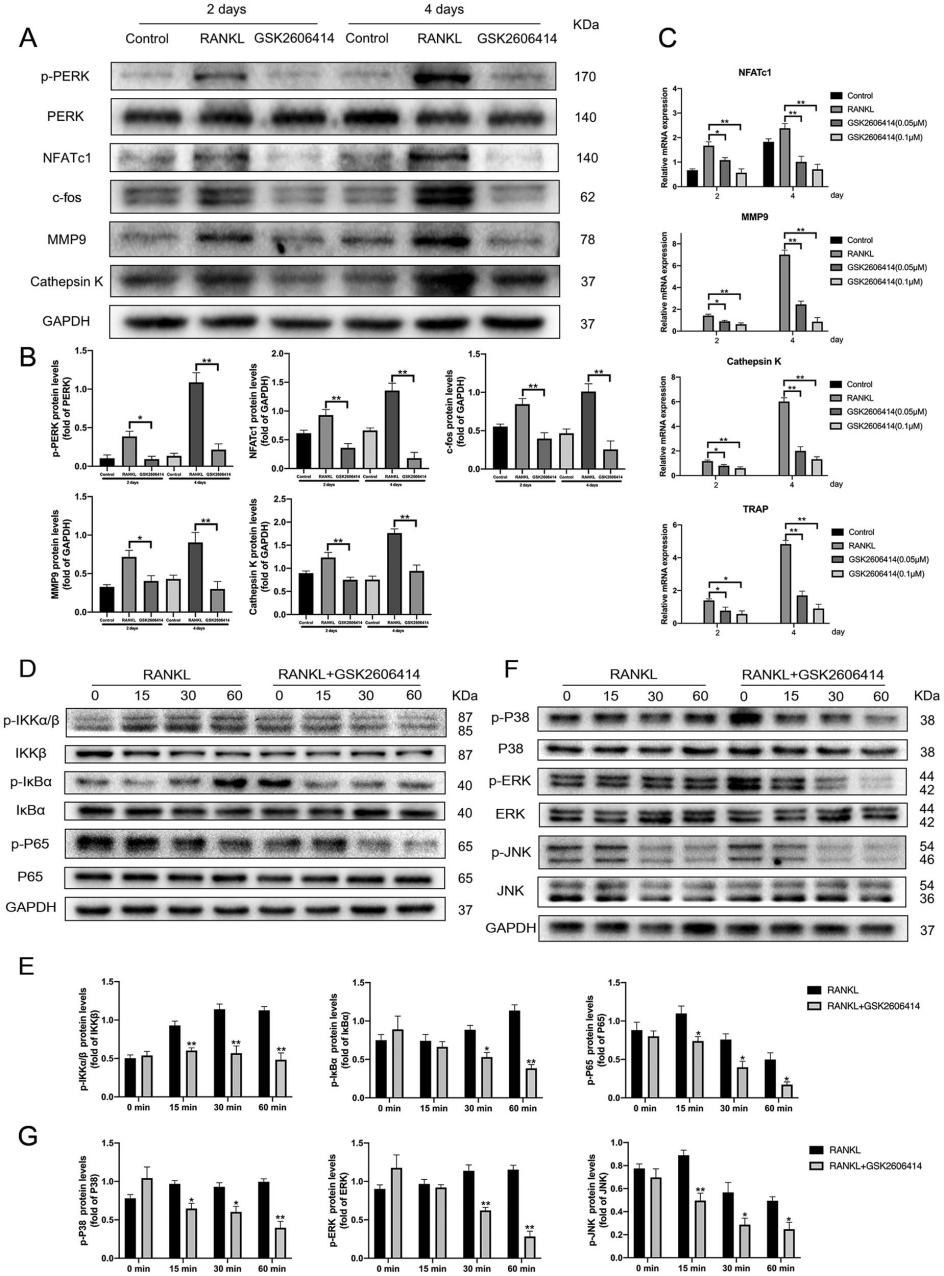
GSK2606414 decreases the expression of osteoclast marker genes and inhibits RANKL-induced NF-κB and MAPKs activation. **a**–**c** BMMs were treated with GSK2606414 (0.1 μM) in the presence of RANKL (100 ng/mL) and M-CSF (30 ng/mL) for 2 or 4 days. p-PERK, PERK and osteoclast-related marker genes proteins expression were measured by western blot at the indicated times and mRNA levels were determined by RT-PCR. **d**–**g** BMMs were starved with a-MEM in the absence of FBS for 12 h, pretreated with or without GSK2606414 for 2 h. After that, BMMs were treated with or without RANKL (100 ng/mL) for the indicated times. The total proteins were extracted and western blot was conducted to detect the expression of NF-κB and MAPK signaling pathways. Densitometric analysis of an immunoblot from three independent experiments; *p < 0.05, **p < 0.01.

On the 2nd and 4th day after RANKL-induced respectively, GSK2606414 obviously inhibited the protein levels of p-PERK, NFATc1, c-fos, MMP9, and Cathepsin K (Fig. 4a, b). Meanwhile, this study also explored whether GSK2606414 inhibited the mRNA expression of the osteoclast-specific genes. The findings demonstrated that GSK2606414 suppresses the transcription level of *TRAP*, *Cathepsin K*, *NFATc1*, and *MMP9* at both early and late stage of osteoclast differentiation (Fig. 4c).

### GSK2606414 inhibited RANKL-induced NF-κB and MAPK activation

Activation of NF-kB and MAPKs play a pivotal role in RANKL-mediated osteoclast differentiation^32,33^. To explore whether GSK2606414 inhibits osteoclastogenesis through inhibition of the MAPK and NF-κB pathways, BMMs were treated with RANKL in the presence of GSK2606414 for 0, 15, 30, and 60 min. As shown in Fig. 4d, e, GSK2606414 attenuated IKKβ, IκBα, and NF-κB p65 phosphorylation. Figure 4f, g showed that treatment with GSK2606414 significantly reduced RANKL-induced phosphorylation of P38 and ERK, but the inhibition of JNK phosphorylation was weaker than that of P38 and ERK.

### GSK2606414 alleviated bone loss in OVX mice

**Fig. 5:**
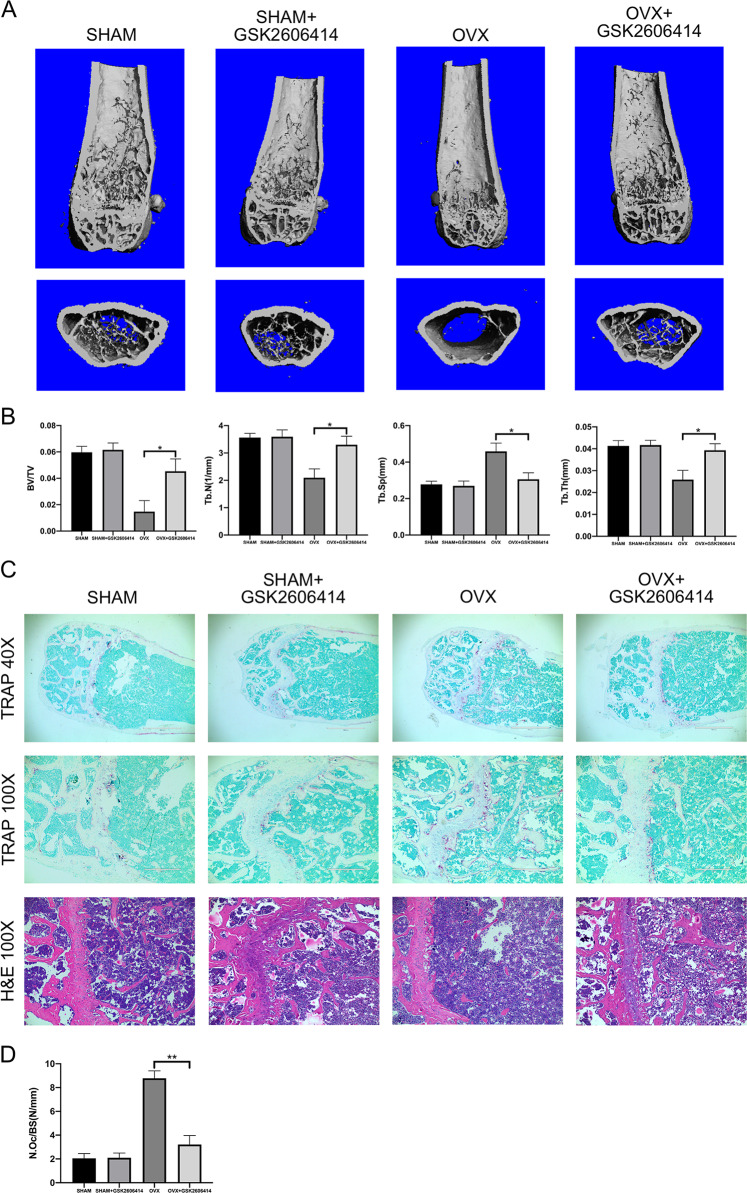
GSK2606414 suppresses OVX-induced bone loss and osteoclast formation. **a**, **b** Micro-CT images of the distal femoral metaphyseal region from the SHAM, SHAM + GSK2606414, OVX, and OVX + GSK2606414 groups. Histograms represent the trabecular structural parameters of the distal femur: BV/TV, Tb.N, Tb.Th and Tb.Sp. Data are presented as means ± SEM, *n* = 8–10 mice per group. *p < 0.05 versus the OVX group. **c**, **d** TRAP and H&E staining were performed on sections of distal femurs. N.Oc/BS of the TRAP staining were calculated. Scale bar (40×) = 1000 μm, Scale bar (100×) = 400 μm. Data are presented as means ± SEM, *n* = 8–10 mice per group. **p < 0.01 versus the OVX group.

We used the OVX mice model to simulate osteoporosis in postmenopausal women. Twelve-week-old female C57BL/6 mice were given GSK2606414 (50 mg/kg) by intragastric gavage the next day after ovariectomy for 6 weeks. Micro-CT was used to analyze the trabecular bone changes in distal femur of different model groups. Compared with SHAM group, OVX + GSK2606414 group demonstrated significantly increase in BV/TV, Tb.N, Tb.Th, while decrease in Tb.Sp. In short, GSK2606414 significantly attenuated trabecular bone loss in OVX mice, and there was no significant difference between SHAM + GSK2606414 group and GSK2606414 group (Fig. 5a, b).

Next, TRAP staining was performed on the femoral sections. Figure 5c, d showed that compared with OVX group, the number of osteoclasts in OVX + GSK2606414 group was significantly increased. As H&E staining shown in Fig. 5c, the trabecular bone density in the growth plate of the distal femur of OVX group became lower. After treatment with GSK2606414, mice trabecular density and thickness raised, this was consistent with the results of micro-CT and TRAP staining.

### GSK2606414 restrained autophagy activation during RANKL-induced osteoclast differentiation

**Fig. 6:**
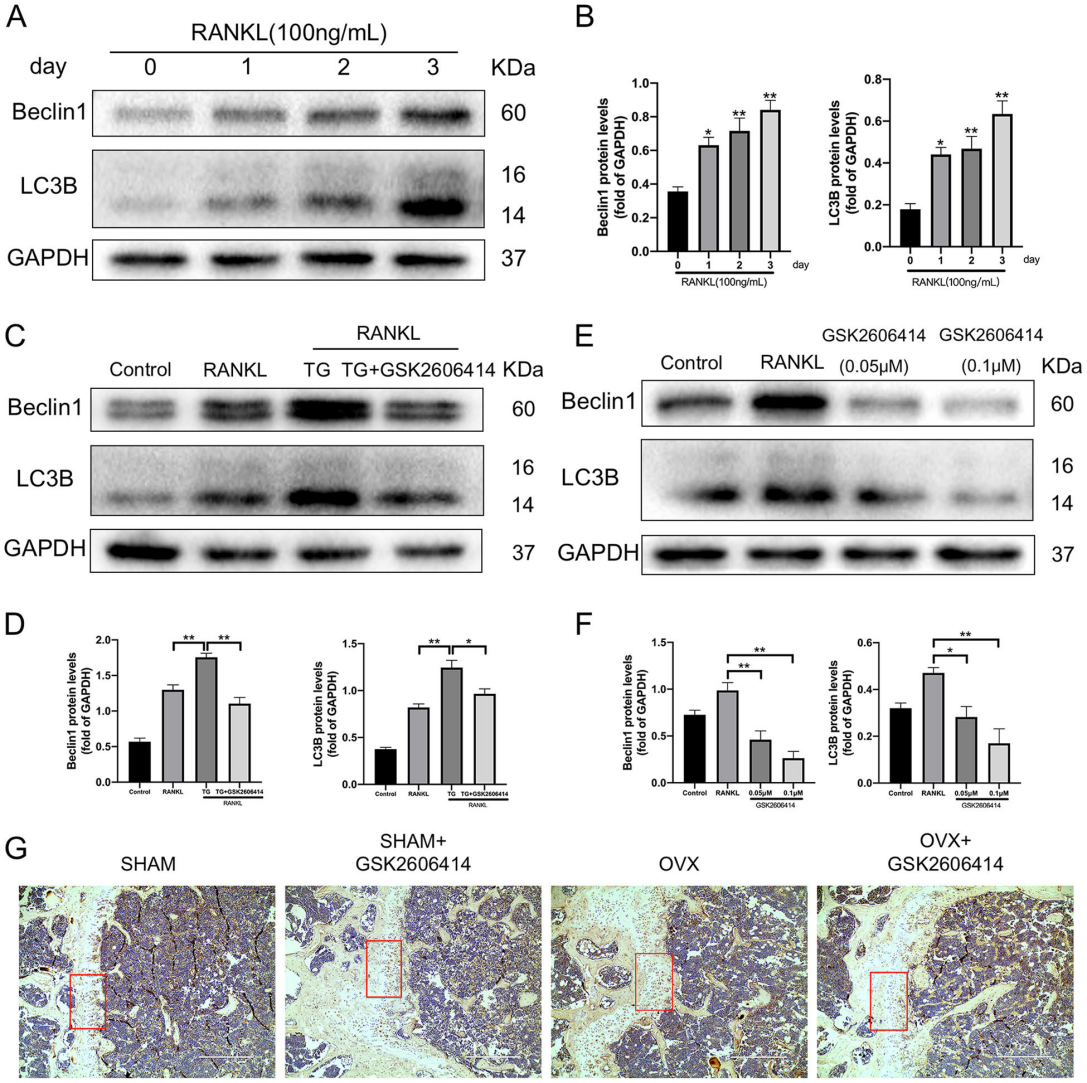
GSK2606414 prevents RANKL-induced increase autophagy-related protein expression and inhibits LC3 expression in OVX mice. **a**, **b** After BMMs were stimulated by RANKL (100 ng/mL) for 0, 1, 2 and 3 days, total proteins were extracted to detect the levels of Beclin1 and LC3B. *p < 0.05, **p < 0.01 versus 0 day group. **c**, **d** Thapsigargin and GSK2606414 were added to osteoclast-induced differentiation medium containing RANKL (50 ng/mL), respectively. Total proteins were extracted on the 4th day and western blot was performed to examine the expression of autophagy-related proteins Beclin1 and LC3B. TG represents thapsigargin in the figure. **e**, **f** BMMs were treated with GSK2606414 (0.05, 0.1 μM) in the presence of RANKL (100 ng/mL) and M-CSF (30 ng/mL) for 4 days, total proteins were extracted and western blot was used to verify the expression of Beclin1 and LC3B. (**g**) C57BL/6 mice were given GSK2606414 (50 mg/kg) by intragastric gavage every 2 days after ovariectomy. Six weeks later, femurs of mice were collected and sectioned, subjected to LC3 immunohistochemical staining. Scale bar = 400 μm, Densitometric analysis of an immunoblot from three independent experiments; *p < 0.05, **p < 0.01.

Autophagy is a pathophysiological process in which eukaryotic cells use lysosomes to clear their damaged organelles and abnormal proteins, and is an important mechanism for maintaining homeostasis^34^. Previous studies reported that the increase of autophagy activity is associated to enhancement of osteoclast differentiation^19^. As seen in Fig. 6a, b, the expression levels of autophagy-related proteins Beclin1 and LC3B during RANKL-induced osteoclastogenesis were increased with time. ER stress could promote autophagy activation through UPR^35,36^. We discovered that during RANKL-induced osteoclast differentiation of BMMs, thapsigargin could significantly up-regulate the expression levels of autophagy-related proteins Beclin1 and LC3B, and PERK inhibitor GSK2606414 could reduce ER stress and inhibit RANKL-induced Beclin1 and LC3B protein expression, respectively (Fig. 6c–f). The results above proved that autophagy was obviously activated during osteoclast differentiation, and the degree of activation is closely related to the intensity of ER stress. Among them, PERK played an important role in the activation of autophagy induced by ER stress during osteoclastogenesis. Similar to these, in experiments in vivo the immumohistochemical staining showed that LC3 expression was increased in the femur sections of mice in OVX group, while it was decreased in the femur sections of mice treated with GSK2606414 (Fig. 6g).

### GSK2606414 inhibited the autophagy flux and formation of autophagosome during osteoclast differentiation

**Fig. 7:**
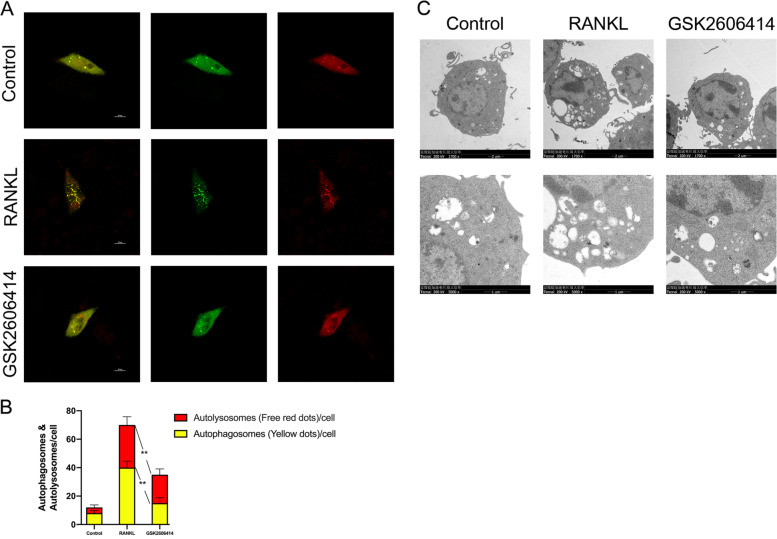
GSK2606414 reduces autophagy flux intensity and autophagosome formation during RANKL-induced osteoclast differentiation. **a**, **b** After infecting BMMs with mRFP-GFP-LC3 tandem fluorescent protein adenovirus, GSK2606414 and RANKL (100 ng/mL) were used to treat BMMs. On the 4th day, the fluorescence was observed with a confocal microscope and quantitative analysis was performed. Scale bar = 10 μm. **c** GSK2606414 and RANKL (100 ng/mL) were used to treat BMMs for 2 days, the formation of autophagosomes in BMMs was observed under transmission electron microscopy. Data are presented as means ± SD of 3 independent experiments; **p < 0.01.

We used mRFP-GFP-LC3 tandem fluorescent protein adenovirus to infect BMMs for labeling and tracking LC3. Red spots represent autophagolysosomes, yellow spots displayed after the red and green fluorescence overlap are autophagosomes, the count of red and yellow spots can be used to determine the intensity of autophagy flow. The results in Fig. 7a, b proved that GSK2606414 could dramatically decrease the intensity of autophagy flux during RANKL-induced osteoclast differentiation. And the electron microscope imaging results were consistent with this, GSK2606414 could suppress the formation of autophagosomes during osteoclastogenesis (Fig. 7c).

### PERK participated in oxidative stress signal transduction during osteoclast differentiation

**Fig. 8:**
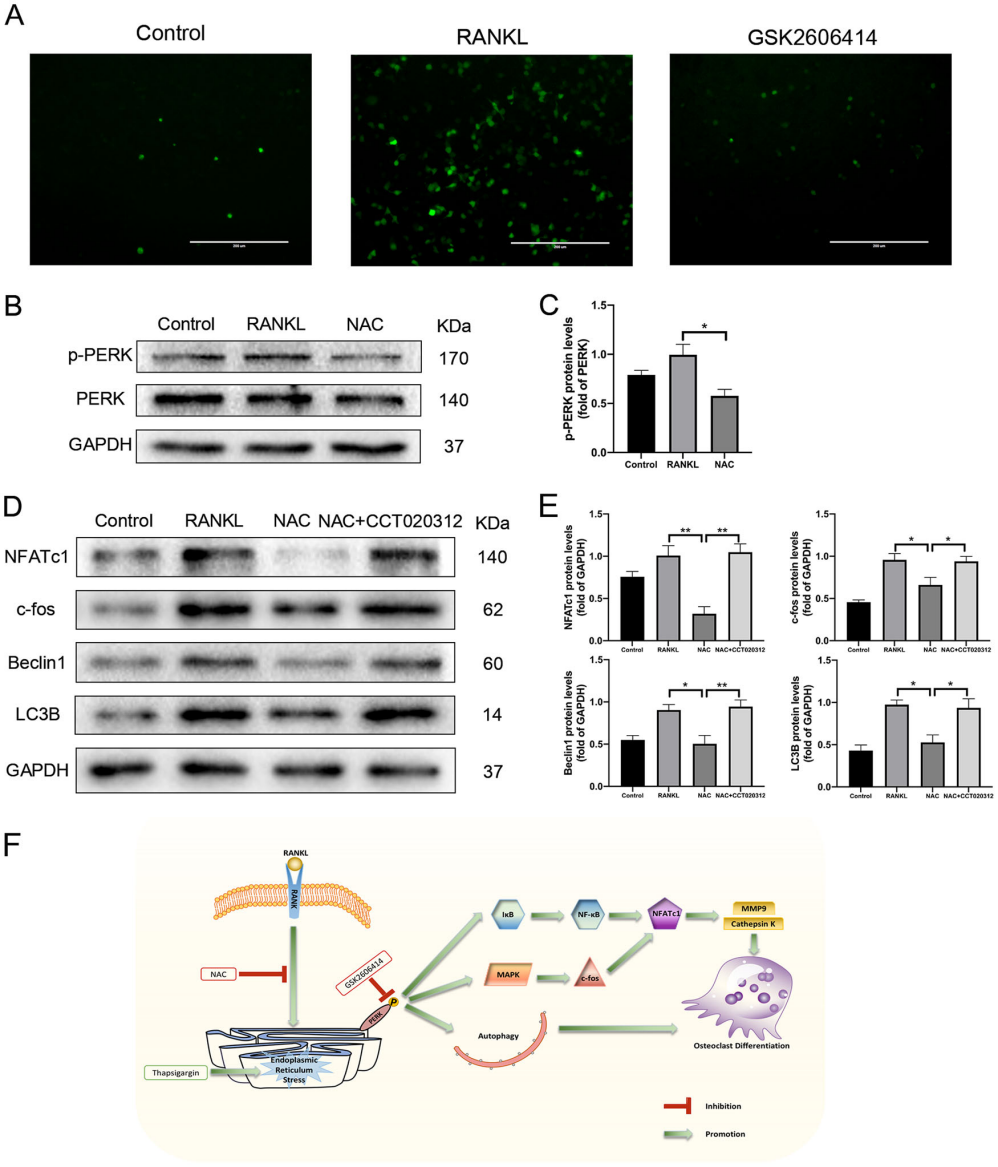
GSK2606414 inhibits RANKL-induced increase of ROS levels, CT020312 reverses the inhibitory effect of NAC on the expression of osteoclast-related and autophagy-related proteins. **a** BMMs were treated with GSK2606414 (0.1 μM) in the presence of RANKL (100 ng/mL) for 4 days, and DCFH-DA probe was used to detect intracellular ROS levels. Scale bar = 200 μm. **b**, **c** RANKL (100 ng/mL) and NAC were used to stimulate BMMs, after 4 days, the total proteins were extracted and western blot was conducted to test the expression of PERK and its phosphorylation level. **d**, **e** BMMs were treated with NAC and PERK activator CCT020312 in the presence of RANKL (100 ng/mL) for 4 days, total proteins were extracted and Western Blot was performed to examine the expression of osteoclast-related proteins NFATc1, c-fos and Autophagy-related proteins Beclin1, LC3B. **f** The schematic model of the hypothesized mechanism by which PERK inhibition affects osteoclast differentiation. Densitometric analysis of an immunoblot from three independent experiments; *p < 0.05, **p < 0.01.

Reactive oxygen species (ROS) include hydrogen peroxide, superoxide anions, and hydroxyl radicals, which are reactive free radicals produced when cells face multiple stimuli. The redox state of cells is closely associated to physiological function of ER, recent studies have exhibited that not only ROS can regulate ER function, but also ER can generate ROS under pathological conditions^37^. ER has a unique oxidative folding environment that promotes endoplasmic reticulum oxidoreductase 1 (ERO1) to acquire the electrons in the reduced protein disulfide isomerases (PDI) and transfer them to dioxygen, thereby generating ROS in the ER^38^. ROS staining revealed that GSK2606414 could restrain the increase of ROS level during RANKL-induced osteoclast differentiation (Fig. 8a). N-acetylcysteine (NAC) is a highly effective antioxidant that reduces osteoclastogenesis by inhibiting intracellular oxidative stress^39^. Through Western blot experiments, we found that NAC could decrease the level of PERK phosphorylation, and reduce the expression levels of osteoclast-related proteins NFATc1, c-fos, and autophagy-related proteins Beclin1, LC3B during RANKL-induced osteoclast differentiation. However, on this basis, treating BMMs with PERK activator CCT020312 reversed the phenomenon that NAC inhibited the expression of osteoclast-related proteins and autophagy-related proteins (Fig. 8b–e). These results proved that oxidative stress might play a vital role as an upstream activator of ER stress during osteoclastogenesis, and PERK was a pivotal molecule in this signal transduction. After being stimulated by oxidative stress, PERK induced autophagy to promote osteoclast differentiation.

## Discussion

The formation of osteoclasts is mainly regulated by two key cytokines, RANKL and M-CSF, and osteoclasts are the only multinucleated giant cells with the capacity of bone resorption function, which is essential for maintaining the homeostasis of the skeletal system^1^. Therefore, in-depth exploration on how to regulate osteoclast differentiation and function has attracted widespread attention.

ER stress is associated with inflammatory responses, metabolic diseases, neurodegenerative diseases, and immunedeficiency diseases^40^. To cope with ER homeostasis disorder and normalize its function, cells are equipped with an evolutionarily conserved stress response, the UPR. Previous studies have shown that the expression of the ER stress marker genes Atf4, Bip, and Edem can be detected up-regulating during osteoclast differentiation, which means that osteoclastogenesis is accompanied by the occurrence of ER stress^41^. Thapsigargin is a classic ER stress activator, it can significantly activate the PERK and IRE1 pathways during osteoclast differentiation^28^. We found that appropriate degree of activation of ER stress is beneficial to osteoclast differentiation. Eun Gyeong Lee et al. reported that the IRE1α/XBP1 pathway could induce the activation of NFATc1, and promote osteoclastogenesis and osteoclast bone resorption activity^42^. Our results showed that PERK pathway were also activated during RANKL-induced osteoclast differentiation, which meant that not only the IRE1 pathway, but also the PERK pathway was closely related to osteoclastogenesis.

PERK (encoded by the *Eif2ak3* gene) is a type I ER transmembrane protein, its protein kinase activity at the carboxyl terminus of its cytoplasmic segment is the main functional region and can phosphorylate eIF2α at protein serine 51 and also autophosphorylate^4,43^. The role of PERK in skeletal development and remodeling has been widely concerned. *Perk* global knockout mice show severe osteopenia, suggesting that PERK is closely associated to bone metabolism^16^. Many researchers have reported the role of the PERK/eIF2α pathway in osteoblast differentiation, for example, salubrinal (selective eIF2α dephosphorylase inhibitor) can up-regulate ATF4 by promoting eIF2α phosphorylation, which cooperates with RUNX2 to regulate the expression of genes related to osteogenesis^44,45^. Whereas, the regulation impact of PERK pathway on osteoclast differentiation is less studied. Jianwen Wei et al. reported that the expression of osteoclast marker genes TRAP and Cathepsin K in bone tissue of *Perk−/−* mice was significantly depressed^16^. Osteoclast precursors that knocked out ATF4 are difficult to differentiate into osteoclasts, and ATF4 can directly accelerate the transcription of *Nfatc1* gene to induce NFATc1 expression and promote osteoclast differentiation^46^. Interestingly, the pharmacological inhibition of eIF2α dephosphatase by salubrinal and guanabenz can inhibit osteoclastogenesis by reducing the expression of NFATc1^47^. From the perspective of the PERK pathway, increasing eIF2α phosphorylation enhances ATF4 activity, these studies reached opposite conclusions on the effect of PERK/eIF2α pathway on osteoclastogenesis. Therefore, we believe that further research is needed to clarify the influence of PERK pathway on osteoclast differentiation.

We have found that inhibiting or silencing PERK has a strong negative regulatory effect on osteoclast differentiation. GSK2606414 significantly inhibited the expression of p-PERK, NFATc1, and c-fos, and down-regulated the levels of osteoclast marker genes MMP9 and Cathepsin K, both in the early stage and in the late stage. The activation of NF-κB and MAPKs plays an irreplaceable role in the formation and function of osteoclasts. Previous studies have proven that silencing PERK down-regulates the phosphorylation of P65 in the cytoplasm by suppressing eIF2α phosphorylation, thereby increasing the radiosensitivity of oropharyngeal cancer cells, and increasing radiation-induced apoptosis and G2/M phase arrest^48^. salubrinal can induce MAPK pathway activation in rat renal tubule ductal epithelial cells^49^. Our results demonstrated that GSK2606414 could inhibit RANKL-induced activation of NF-κB and MAPK signaling pathways. In addition, micro-CT results of experiments in vivo further indicated that inhibition of PERK could ameliorate bone loss in OVX mice.

During bone remodeling, the fusion of lysosome and plasma membrane plays a key role in osteoclast degradation of the bone matrix. Autophagy is involved in regulating the biological functions of lysosomes and is essential for maintaining the homeostasis of the cellular environment^50^. Autophagy/lysosomal inhibitor chloroquine reduces osteoclast differentiation and formation by suppressing TRAF3 degradation^51^; TRPV4 overexpression in RAW264.7 cells up-regulates the expression levels of autophagy-associated proteins (LC3B and Beclin1) to accelerate osteoclast formation, and autophagy inhibitor 3-methyladenine reverses this effect^52^. The formation and function of osteoclasts require the participation of autophagy-related proteins, suggesting that the autophagy plays a marked role in osteoclastogenesis^53^. We found that the expression of Beclin1 and LC3B increases with time during RANKL-induced osteoclast differentiation. It can be understood that autophagy activation is of great significance for osteoclastogenesis.

ER stress restores protein folding ability and intracellular homeostasis by initiating UPR, and autophagy is important for eliminating abnormally accumulated proteins in cells. Studies have exhibited that the three signal molecules PERK, IRE1, and ATF6 that mediate UPR are involved in regulating autophagy process^15^. Among them, PERK is involved in the upregulation of many autophagy-related proteins, including ATG6, LC3B, ATG5, and Beclin1. Intermittent hypoxia can cause pancreatic β-cells apoptosis, but LC3B and ATG5 transcription is enhanced in the cells through PERK/eIF2α/ATF4 pathway to activate autophagy and cause protective response against apoptosis^54^. We found that thapsigargin significantly increased the expression of Beclin1 and LC3B during osteoclast differentiation, indicating that the degree of autophagy activation is closely associated to the intensity of ER stress, while the PERK inhibitor GSK2606414 occupied a significant negative regulation impact on this effect. Besides, GSK2606414 attenuated the intensity of autophagy flux and reduced autophagosome formation during RANKL-induced osteoclastogenesis.

ROS is involved in the regulation of physiological intracellular redox status and is related to cell fate such as proliferation, differentiation, and apoptosis. ROS plays a crucial regulatory role in the process of bone remodeling, it restrains osteoblast activation and mineralization by increasing the ratio of RANKL/OPG, and accelerates osteoclast differentiation and bone resorption^55,56^. During RANKL-induced osteoclastogenesis, a signal cascade involving TRAF6, Rac1, and Nox1 stimulates the production of intracellular ROS, and ROS acts as intracellular signaling medium for cell differentiation into osteoclasts^57^. The production of ROS may induce ER calcium depletion and accumulation of abnormally oxidatively modified proteins, then as a pathway of ER stress, PERK pathway is activated, restores ER homeostasis by reducing the protein load in ER and activating NRF2-induced antioxidant pathway^58^. Since cell differentiation involves the renewal of proteins, it is highly related to the process of cell self-digestion (eg. autophagy)^59^. We found that the antioxidant NAC inhibited the activation of PERK and down-regulated the expression of autophagy-related proteins during osteoclast differentiation, and the use of PERK activators reversed this effect. We speculate that oxidative stress caused by ROS during osteoclastogenesis results in ER stress, and autophagy is activated by PERK to maintain cellular homeostasis.

In conclusion, this study suggested that PERK was a key target for regulating osteoclast differentiation and function. Our results demonstrated that GSK2606414 inhibits bone loss in OVX mice, and autophagy is involved in the regulation of osteoclast differentiation by PERK. We figure that during osteoclast differentiation, oxidative stress leads to ER stress, which in turn activates autophagy. Our conclusions are intended to provide new clues to the molecular mechanisms of osteoclastogenesis.

## Materials and methods

### Reagents and antibodies

Thapsigargin and CCT020312 were purchased from MedChemExpress (New Jersey, United States). Hydroxypropyl methylcellulose (HPMC) and DMSO were acquired from Sigma-Aldrich (St. Louis, United States). GSK2606414 and antioxidant N-acetylcysteine (NAC) were obtained from Selleck (Houston, United States). Thapsigargin, CCT020312, GSK2606414, and NAC were dissolved in DMSO for in vitro use. GSK2606414 was dissolved in solution containing 0.5% HPMC and 0.1% Tween-80 for in vivo use. Recombinant mouse RANKL was bought from R&D Systems (Minneapolis, United States). Recombinant mouse M-SCF was bought from PeproTech (Rocky Hill, United States). Rabbit antibodies against Beclin1 (#3495), LC3B (#3868), BIP (#3177), elF2α (#5324), p-elF2α (#3398), NFATc1(#8032), p-ERK (#4370), ERK (#4695), P38 (#8690), p-P38 (#4511), JNK (#9252), p-JNK (#4668), p-IκBα (#2859), IKKβ (#8943), p-IKKα/β (#2697), P65 (#8242), p-P65 (#3033) and mouse antibody against IκBα (#4814) were obtained from Cell Signaling Technology (Boston, United States). Rabbit antibody against PERK (ab229912) was purchased from Abcam (Cambridge, United Kingdom). Rabbit antibody against p-PERK (DF7576) was acquired from Affinity Biosciences (Ohio, United States). Rabbit antibodies against Cathepsin K (11239-1-AP) and MMP9 (10375-2-AP) and mouse antibody against GAPDH (60004-1-lg) were bought from Proteintech (Rosemont, United States). Rabbit antibody against c-Fos (H-125) was obtained from Santa Cruz (CA, United States).

### Cell culture

Bone marrow-derived macrophages (BMMs) were obtained from the femurs and tibias of 6-week-old female C57BL/6 mice as described previously^60^. Concisely, bone marrow cavities of isolated femurs and tibias were flushed with culture medium, and repeated 2–3 times until the diaphysis was completely white, the cell suspension was collected. The obtained cell suspension was carefully filtered with a 70 μm filter, and then centrifuged at 1200 rpm for 5 mins, then the cells were resuspended with erythrocyte lysis buffer. After standing for 1 min, the centrifugation operation (1200 rpm, 5 mins) was repeated. The cells which is the BMMs needed for our experiments were resuspended in α-MEM complete medium containing 30 ng/mL M-CSF, 10% FBS (Gibco, United States), 100 μg/mL streptomycin and 100 μg/mL penicillin.

### Cell counting kit-8 assay

Cell viability and proliferation were assessed using CCK 8 (Boster, Wuhan, China). BMMs were seeded in 96-well plates and treated with thapsigargin (0, 0.05, 0.1, and 0.2 nM) or GSK2606414 (0, 0.01, 0.05, and 0.1 μM). At the specified time point, medium containing 10% CCK 8 solution was added to each well, and then the cells incubated in darkness for an hour. The absorbance was measured at 450 nm using ELX800 absorbance microplate reader (Bio-Tek, Winoosk, United States).

### In vitro osteoclastogenesis assay

BMMs were seeded in 96-well plates (1 × 10^4^ cells/well), cultured with M-CSF, RANKL, and treated with different reagents (thapsigargin, GSK2606414 and PERK-siRNA) for 4 days. TRAP staining was performed with TRAP staining kit (Sigma-Aldrich) according to the manufacturer’s protocol. Images were acquired by EVOS FL auto cell image system (Life Technologies, United States). TRAP-positive multinucleated giant cells containing more than 3 nuclei are considered as osteoclasts.

### Western blot

Western Blot was conducted as described previously^61^. After treated, BMMs were lysed by using RIPA Buffer (Boster) containing phosphatase inhibitor and protease inhibitor to extract total protein. Equal quality of protein (25 μg) was added to 8% or 10% SDS-polyacrylamide gel. After electrophoresis, the protein was transferred to PVDF membranes (Millipore, Billerica, United States) and blocked for 1 h with 5% BSA in TBST. The PVDF membranes were then placed in respective primary antibodies solution to incubate overnight (over 16 h) at 4 °C. Next, the PVDF membrane was incubated with secondary antibodies diluted in 5% BSA solution for 1 h. The immunoreactive proteins were determinated with ECL (Boster) and the photographs were taken with ChemiDoc^TM^ XRS + system (Bio-Rad, United States).

### Small interfering RNA transfection

The small interfering RNA (siRNA) used in this study was purchased from Ribbio Biological (Guangzhou, China). siRNA-1: CGGGAAAACGGTTCTGAGA, siRNA-2: CCACAGACATCA TTGAAAA, siRNA-3: GAGTTCATCTGGAACAAAA. Transfection complex solution was prepared according to manufacturer’s protocol. BMMs were transfected with siRNAs (20 mM) using siRNA Transfection Reagent with M-CSF (30 ng/mL). After incubating with the siRNA mix (100 nM) for 6 h, the cells were cultured with medium in the presence of RANKL. The siRNA transfection was performed every 72 h.

### Bone resorption assay

BMMs were seeded in Osteo Aaasy Surface plate (Corning, NY, United States) at a density of 2 × 10^4^ cells/well, and cultured with α-MEM complete culture medium (containing 100 ng/mL RANKL and 30 ng/mL M-CSF) until each group has mature osteoclasts, then cells were treated with different concentrations of GSK2606414 or siRNA. After additional 4 days, the plate was washed with PBS and 5% sodium hypochlorite. Images were photographed under inverted phase contrast microscope and imaging system (Nikon, Japan). Bone resorption area was calculated by using Image J software.

### Actin ring and DAPI staining

BMMs were seeded in 96-well plates at a cell density of 1 × 10^4^ cells/well, cultured in α-MEM medium containing RANKL and M-CSF, and F-actin ring and DAPI staining were performed 4 days after treatment. After fixing with immunol staining fix solution (Beyotime, Shanghai, China) at room temperature for 15 mins, the cells were washed three times with immunol staining wash buffer (Beyotime) for 5 mins each time and treated with phalloidin at room temperature for 60 mins. DAPI staining was then performed and incubated in the dark at room temperature for 5 mins. Images were photographed under a fluorescence microscope (EVOS FL auto cell image system, Life Technologies, United States).

### Reverse transcription and quantitative PCR

Total RNA was extracted using E.Z.N.A Total RNA extracting kit (Omega, United States), and reverse transcription was performed using ReverTra Ace qPCR RT kit (Toyobo, Osaka, Japan) based on the measured total RNA concentration. Reverse transcription and quantitative PCR (RT-qPCR) was conducted by RT-qPCR kit (Toyobo) and Bio-Rad Q5 instrument (Bio Rad). The primers sequences involved were as follows (F, forward; R, reverse):

GAPDH, F 5′-ACCCAGAAGACTGTGGATGG-3′ and R 5′-CACATTGGGGGTAG GAACAC-3′; NFATc1, F 5′-GACCCGGAGTTCGACTTCG-3′ and R 5′-TGACACT AGGGGACACATAACTG-3′; TRAP, F 5′-CACTCCCACCCTGAGATTTGT-3′ and R 5′-CATCGTCTGCACGGTTCTG-3′; Cathepsin K, F 5′-GAAGAAGACTCACCA GAAGCAG-3′ and R 5′-TCCAGGTTATGGGCAGAGATT-3′; MMP9, F 5′-CTGGA CAGCCAGACACTAAAG-3′ and R 5′-CTCGCGGCAAGTCTTCAGAG-3′.

### Animal model

C57BL/6 female mice (12 weeks old) purchased from the Animal Experiment Center of Tongji Hospital were used for our study. The animal study was authorized by the Ethics Committee on Animal Experimentation of Tongji Medical College (Wuhan, China).

40 mice were randomly divided into four groups (*n* = 10), ovariectomy (OVX) or sham surgery was performed as previously described^62^. The mice were anesthetized by intraperitoneal injection with 1% sodium pentobarbital solution (50 mg/kg), and the bilateral ovaries were removed through a dorsal approach. After finding the ovary, the mice in the sham group were returned to the abdominal cavity without resection. Two days after the operation, GSK2606414 was administered to mice by intragastric gavage. GSK2606414 (50 mg/kg) or vehicle (solution containing 0.5% HPMC and 0.1% Tween-80) were administered every 2 days for 6 weeks.

### Micro-computed tomography (μ-CT)

Bilateral femurs from each group of mice were removed, and then the femurs were placed in 4% paraformaldehyde for 24 h. The micro-CT (Scanco Medica, Switzerland) scan was conducted and the parameters are 100 kV, 98 µA, and the scan interval is 10.0 µm. We used micro-CT software to analyze three-dimensional reconstruction, and results parameters included trabecular number (Tb.N), bone volume/tissue volume (BV/TV), trabecular thickness (Tb.Th), and trabecular separation (Tb.Sp).

### Histomorphometric analysis

After 24 h of fixation, the femurs were decalcified with 10% EDTA solution. The decalcification solution was changed every 2 days until the syringe needle could easily penetrate the cortical bone. Next, paraffin embedding was performed and 5 μm thick sections were made and dewaxed. Hematoxylin-eosin (H&E), tartrate-resistant acid phosphatase (TRAP), and immunohistochemical staining were performed following the standard protocols. After staining, the slices were observed using a light microscope and imaging system (EVOS FL auto cell image system).

### Transmission electron microscope

Transmission electron microscope (TEM) is an important method to observe autophagosomes because it can directly visualize the internal structure of cells. The procedure was performed following the standard protocols. In brief, cells were washed with PBS, and then fixed by electron microscope fixing solution (Powerful biology, Wuhan, China), dehydrated with different concentrations of alcohol and acetone and then embedded in epoxy resin. Ultrathin sections were stained with uranyl acetate (E. Merck, Darmstadt, Germany) and lead citrate (Sigma Aldrich). Transmission electron microscope (JEM-100CXII, Japan) was used for observation.

### Autophagy double-labeled adenovirus (mRFP-GFP-LC3) transfection

The mRFP-GFP-LC3 adenovirus was purchased from Hanbio Biotechnology (Shanghai, China). BMMs were seeded into the cell plate and ensured that the cell density will be between 50 and 70% on the next day. Formation of autophagolysosome by the fusion of lysosome and autophagosome results in change of pH, then GFP fluorescence quenchs (GFP fluorescent protein is sensitive to acidic environment). At this time, only red fluorescence can be observed under a confocal microscope. Therefore, whether the lysosome fuses with autophagosome to form autophagolysosome can be determined by observing the fluorescence change of GFP. After merging in the microscopic imaging, red spots indicate autophagolysosomes, yellow spots displayed by red-green fluorescence overlap are autophagosomes. The count of red and yellow spots can be used to exhibition the level of autophagy.

### Reactive oxygen species detection

ROS detection was conducted as described previously^62^. 29,79-dichlorofluorescin diacetate (DCFH-DA, Sigma-Aldrich) is a fluorescent dye that can penetrate cell membranes. DCFH-DA was diluted as 1: 1000 in serum-free culture medium (final concentration was 10 μM). Appropriate volume of DCFH-DA solution was added to cells, and incubate at 37 °C in the dark for 30 mins. Next, the cells were washed three times with serum-free medium to remove excess DCFH-DA. The level of ROS was observed by fluorescence microscope.

### Statistical analysis

All experiments in this study were repeated at least three times independently. Results were expressed as means ± standard difference (SD). Comparisons between the two groups were performed using student’s *t*-test, and ANOVA followed by a Tukey test was used for multiple comparisons. *p < 0.05 and **p < 0.01 indicated significantly difference, NS represents there is no statistical difference.

## Supplementary information

supplementary Figure Legends (clean version)

Supplementary Figure 1
