# Supplementary material for: PERK controls bone homeostasis through the regulation of osteoclast differentiation and function
Source: Cell Death Dis. 2020 Oct 13;11(10):847. doi: 10.1038/s41419-020-03046-z (PMC7554039; doi:10.1038/s41419-020-03046-z)
Supplement: Supplementary file 1 — supplementary Figure Legends (clean version) [file 41419_2020_3046_MOESM1_ESM.docx]

Supplementary Material

**PERK controls bone homeostasis through the regulation of osteoclast differentiation and function**

**Jiachao Guo****^1^, Ranyue Ren^1^, Kai Sun^1^, Xudong Yao^1^, Jiamin Lin^1^, Genchun Wang^1^, Guo Zhou^1^, Tao Xu^2^ and Fengjing Guo^1^**

**1** Department of Orthopedics, Tongji Hospital, Tongji Medical College, Huazhong University of Science and Technology, Wuhan, China.

**2** Department of Rehabilitation, Tongji Hospital, Tongji Medical College, Huazhong University of Science and Technology, Wuhan 430030, China

These authors contributed equally: Jiachao Guo, Ranyue Ren

793527829@qq.com (JG); [renranyue@hust.edu.cn](mailto:renranyue@hust.edu.cn) (RR);

[602983321@qq.com](mailto:602983321@qq.com) (XY); [1085844308@qq.com](mailto:1085844308@qq.com) (KS);

[1428975034@qq.com](mailto:1428975034@qq.com) (JL); [455703865@qq.com](mailto:455703865@qq.com) (GW);

1178519539@qq.com (ZG)

**Correspondence to:**

Prof. Tao Xu Prof. Fengjing Guo

Tel: +86-27-8366-5238 (TX) +86-27-8366-5218 (FG)

Fax: +86-27-8366-2640 (TX) +86-27-8366-3670 (FG)

[xutao0101@yeah.net](mailto:xutao0101@yeah.net) m201575777@hust.edu.cn

Supplementary Material Figure Legend

**Figure S1**. (A-B) The concentrations of thapsigargin and GSK2606414 used in this study did not affect the viability of BMMs. BMMs were seeded in 96-well plates and adhered, culture medium was changed with different concentrations of thapsigargin or GSK2606414 every day, and the cell viability was detected by CCK-8 assay at the corresponding time point. The influences of thapsigargin and GSK2606414 on the viability of BMMs were showed. (C-D) Verification of the knockdown ability of PERK-siRNA to PERK protein. Three different fragments of PERK-siRNAs were used to treat BMMs, western blot was used to detect the expression of PERK protein. It was found that siRNA-3 had the strongest knockout ability on PERK. Densitometric analysis of an immunoblot from three independent experiments; *p < 0.05, **p < 0.01.
