# Supplementary figures and images for: PERK controls bone homeostasis through the regulation of osteoclast differentiation and function
Source: Cell Death Dis. 2020 Oct 13;11(10):847. doi: 10.1038/s41419-020-03046-z (PMC7554039; doi:10.1038/s41419-020-03046-z)

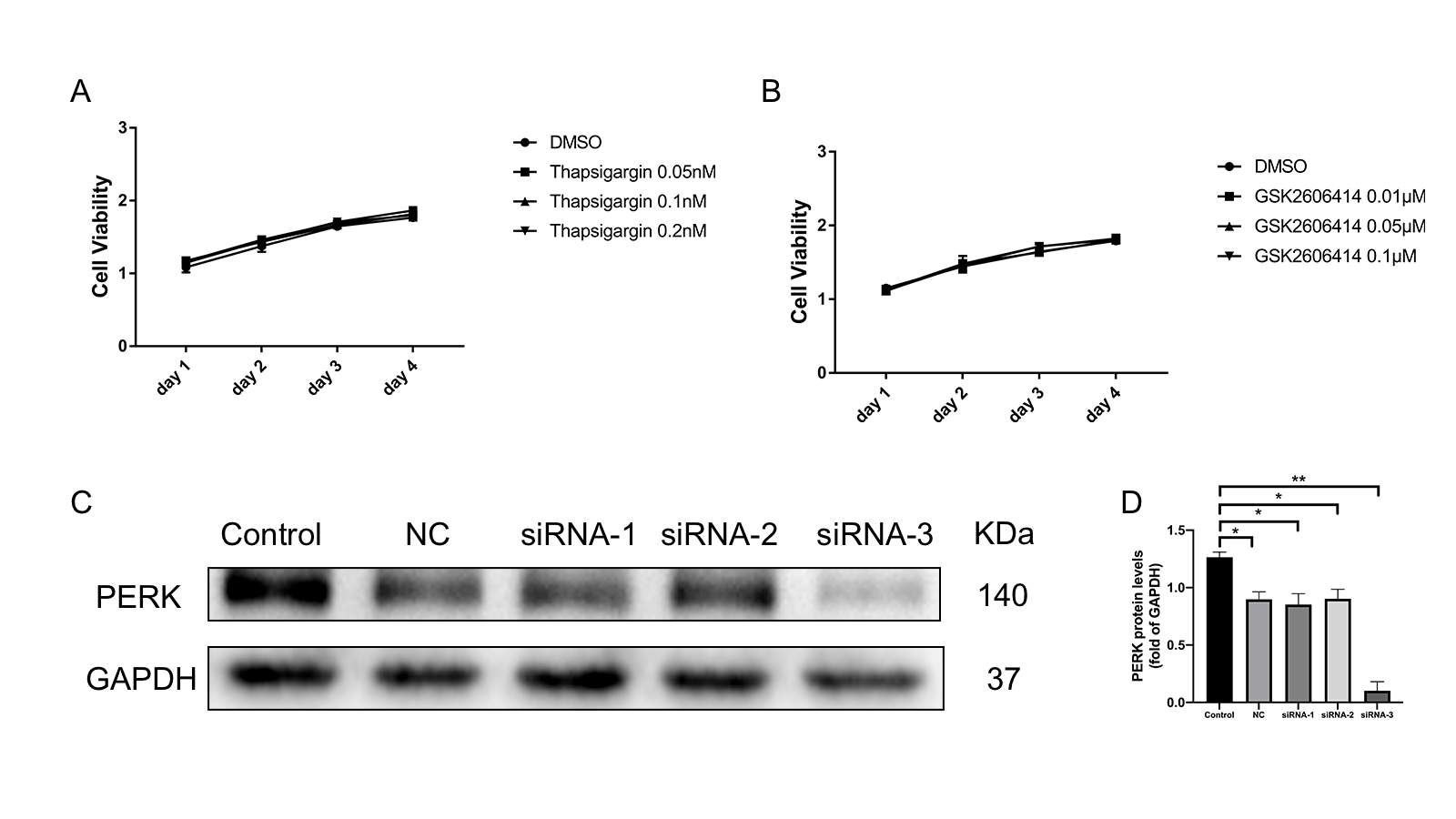

Supplement: Supplementary file 2 — Supplementary Figure 1 [file 41419_2020_3046_MOESM2_ESM.tif]
